# Formula for the progressive reintroduction of fat into breast milk in infants with chylothorax

**DOI:** 10.1002/jpr3.70235

**Published:** 2026-08-31

**Authors:** Sonia María Alonso‐Clemente, Ana Concheiro‐Guisan

**Affiliations:** ^1^ Department of Nutrition and Dietetics Álvaro Cunqueiro University Hospital Vigo Spain; ^2^ Department of Pediatrics Álvaro Cunqueiro University Hospital and Galicia Sur Health Research Institute Vigo Spain

**Keywords:** breastfeeding, lymphatic leak, newborn, skim milk

## Abstract

Chylothorax in infants is a potentially life‐threatening condition, often resulting from injury during cardiac surgery. Traditionally, breastfeeding is discontinued. Recently, feeding with defatted human milk has been safely introduced. However, methods for fat reintroduction are not standardized. We developed a mathematical formula to facilitate individualized, progressive reintroduction of fat into skimmed breast milk. Fat and protein content were analyzed by Fourier‐transform infrared spectroscopy (FTIR). Defatting was performed by centrifugation at 3000 rpm at 2°C for 15 min. The formula considers: fat concentration of unmodified human milk and defatted milk, target fat concentration, and milk volume. Clinical application in a premature infant showed effective fat reintroduction (0.2 g/dL every 48 h), with no recurrence of chylothorax. Some factors influenced milk defatting. Breast milk provides important immunological and functional benefits. Our formula supports safe administration and can assist in designing further studies to determine optimal daily fat increments.

## Abstract (highlights)

**What is Known:**

- Breastfeeding is safe in infants with lymphatic leak.
- Defatting (skimming) of breast milk is required for this nutritional management.
- There is currently no well‐defined protocol for the reintroduction of fat into skimmed breast milk once the chylothorax has resolved.

**What is New:**

- A mathematical formula has been developed to facilitate fat reintroduction.
- This formula allows a safe and individualized approach for each infant.
- Both clinical practice and future research aimed at further exploring these issues may benefit from the use of this formula.
- The importance of certain factors that can influence degreasing is highlighted.

## 1 INTRODUCTION

Chylothorax is the accumulation of lymph in the pleural space. In neonates, it may be congenital or acquired, with congenital cases usually caused by lymphatic vessel abnormalities and acquired forms often postoperative.^1^, ^2^, ^3^ Congenital chylothorax (CC) is rare, approximately 1 in 5000–10,000 live births, with survival rates between 30% and 70% depending on etiology, gestational age, and comorbidities.^1^

Lymph extravasated in chylothorax contains fats, proteins, lymphocytes, antibodies, and complement proteins,^4^ and its loss can cause malnutrition, dehydration, and increased infection risk. Treatment may require pleural drainage, pharmacological therapy with somatostatin analogs, or thoracic duct ligation for persistent effusions.^5^

The cornerstone of management is dietary modification to reduce lymph production and allow lymphatic healing.^6^, ^7^ Traditionally, enteral nutrition is replaced with parenteral nutrition or medium‐chain triglycerides (MCT)‐based formula until pleural output decreases. Human milk was historically contraindicated due to fat content.^1^, ^2^, ^3^, ^6^, ^7^

Since the 2000s, defatted human milk has been safely administered,^8^, ^9^, ^10^, ^11^, ^12^ with studies showing growth outcomes comparable to MCT‐based formulas when fortification is adequate.^9^, ^10^, ^11^ Cold centrifugation is the most common defatting method, with fat content confirmed by creamatocrit or more recently by Fourier‐transform infrared (FTIR) spectroscopy.^13^, ^14^

Despite standardization of defatting, timing and method of fat reintroduction remain poorly defined.^12^, ^15^ The primary aim of this work is methodological, specifically the development of a mathematical formula to facilitate the individualized reintroduction of fat into skimmed breast milk. The clinical case included is intended only as an example of applicability, and conclusions derived from a single real‐case application should therefore be interpreted with caution.

## 2 METHODS

### 2.1 Ethics statement

The submitted study was approved by the Research Ethics Committee of Pontevedra–Vigo–Ourense (code 2016/559) and informed consent was obtained from research subjects, including all milk donor mothers and the parents (legal guardians) of the infant whose case is specifically described. Period of study: June 2024 to June 2025.

### 2.2 Mathematical formula design

A mathematical formula was developed to guide the controlled reintroduction of fat into skimmed breast milk. The formula accounts for the initial milk fat content, target fat concentration, total feeding volume, and the infant's individual nutritional requirements, enabling a safe, stepwise, and individualized approach. The variables and parameters used in the formula were defined as follows:

- **C1:** Fat content in defatted milk (target ~0 g/dL)
- **C2:** Fat content in unmodified human milk. It varies among lactating women. It has been reported on average between 2.52 and 4.11 g/dL in previous works^13^
- **C3:** Target fat concentration for reintroduction. Considering the mean fat content of human milk, increments should not exceed 10% of the lower average fat content in human milk (0.2 g/dL).
- **V:** Volume of milk to be administered

**Figure 1:**
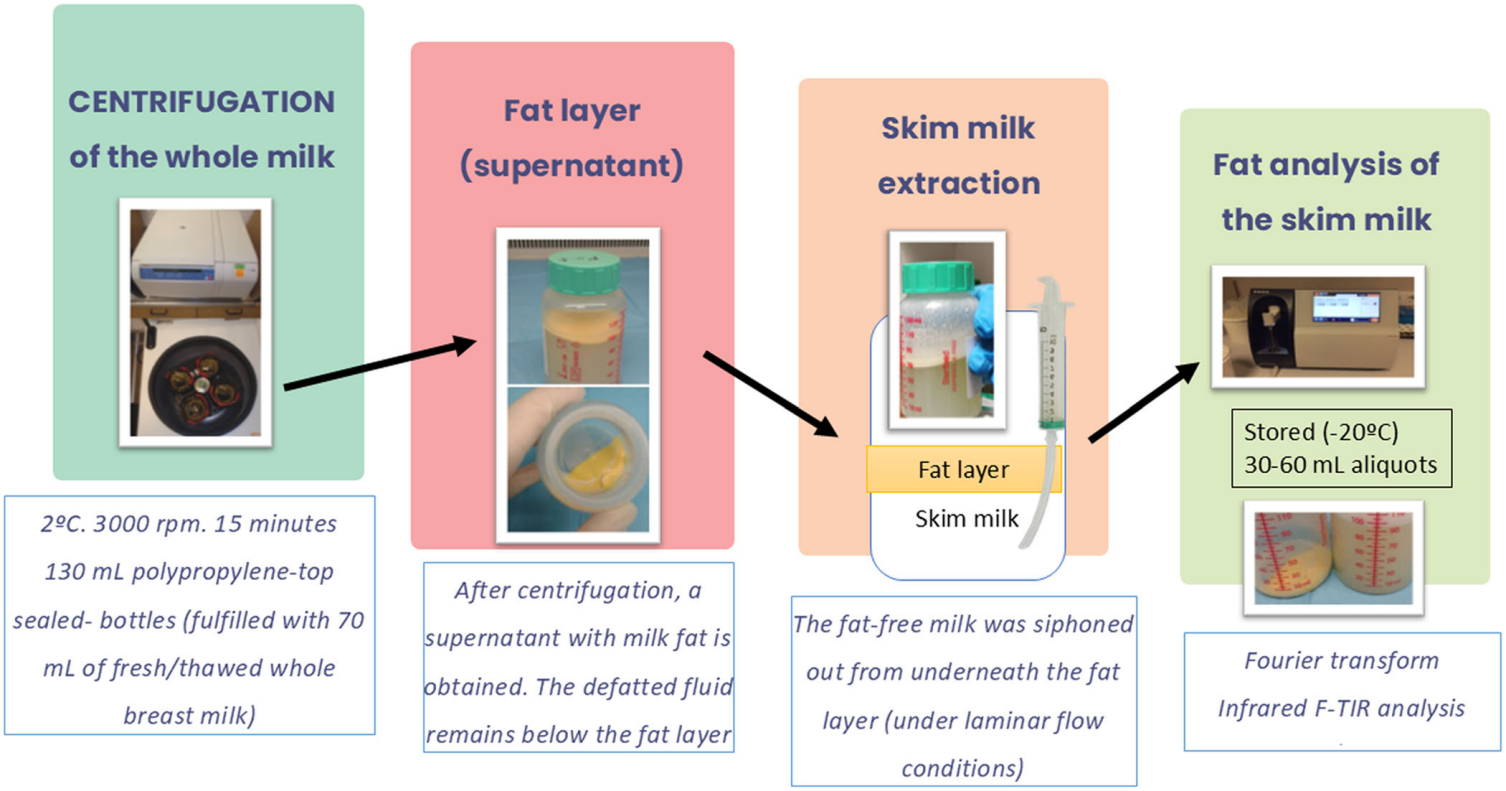
Flowchart of the standardized human milk defatting process.

We selected a pool of samples from all women actively donating to our milk bank between June 2024 and May 2025 for the purpose of fat removal and subsequent quantification of fat concentration. All participants met the eligibility criteria for breast milk donation. Women were at different lactation stages. Half of the samples were chosen from mothers of preterm infants. Each woman provided one or two donated samples. Defatted milk was obtained by centrifugation at 3000 rpm, 2°C, for 15 min.^13^, ^14^ Milk was stored in polypropylene containers of 60 and 130 mL (larger containers were observed to deform during centrifugation). In 130 mL aliquots, the milk volume did not exceed 70 mL. Fat was removed via aspiration with a cannula and syringe as this method has been described as the most effective for achieving a milk sample with minimal fat content, approaching zero.^13^, ^14^ Milk fat and protein content were measured using FTIR spectroscopy (Foss Milkoscan, Hillerød, Denmark). Figure 1 summarizes the defatting process. Factors influencing the defatting process were determined by the analysis of different samples under distinct conditions.

### 2.3 Clinical application

The practical application of the formula in a real‐world clinical scenario was proposed. Fat reintroduction was performed using fresh or frozen unskimmed maternal milk, which was thawed and then recombined with defatted milk at room temperature. Multiple measurements of fat concentration using spectrometry, correlated with the calculated values from the formula, were performed throughout the incremental fat reintroduction process.

## 3 RESULTS

### 3.1 Mathematical formula

The reference standardized procedure^13^, ^14^ was applied to 122 milk samples from 54 different women, with fat content ranging from 1.59 to 3.58 g/dL, ranges lower than those described in other research studies.^13^

Factors improving defatting included:

- Centrifugation speed (3000 vs. 2000 rpm)
- Lower baseline maternal milk fat content (< 2 g/dL)
- Aliquot size (130 mL vs. 60 mL)

Mean fat content after defatting under these conditions was 0.008 g/dL, with no significant change in protein (2.62 vs. 2.56 g/dL).

A higher average fat content was observed in milk from mothers of preterm infants (3.01 g/dL) and in those with lactation longer than 6 months (2.64 g/dL) compared to mothers with shorter lactation periods (2.57 g/dL).

The formula calculates the volume of whole milk to be added to defatted milk for safe, progressive fat reintroduction. It includes four main components:

- **C1:** Fat content in defatted milk (target ~0 g/dL)
- **C2:** Fat content in unmodified human milk. It varies among lactating women.
- **C3:** Target fat concentration for reintroduction
- **V:** Volume of milk to be administered
- **V2:** the volume of unmodified milk to be added, defined as *V* × *w*_2_, and is mixed with the defatted milk (V1) for fat reintroduction
- The resulting formula is ***w***_**2**_ = **(*C*3** − ***C*1)/(*C*2** − ***C*1)**

### 3.2 Clinical application

**Figure 2:**
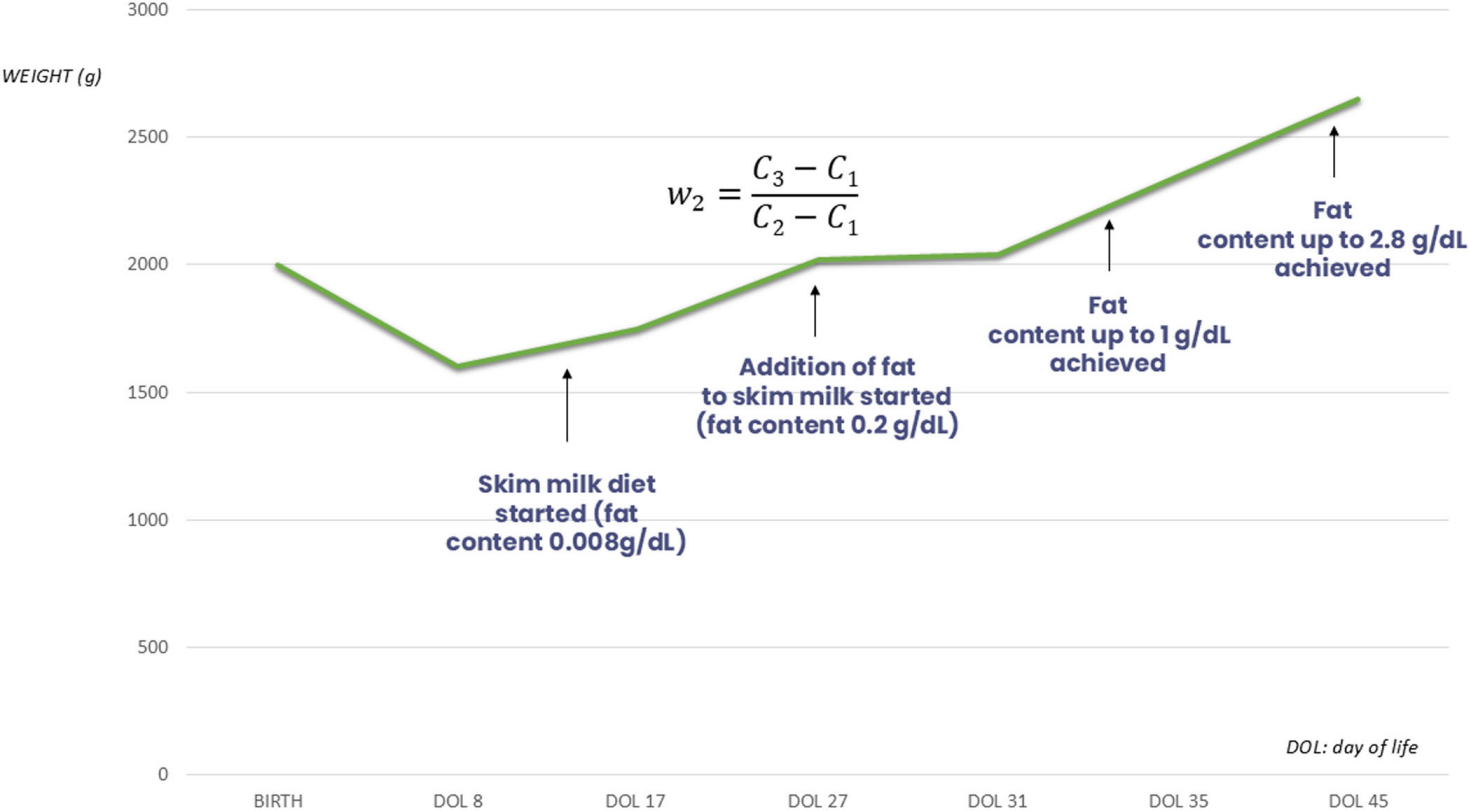
Growth curve of the infant during defatted milk feeding and fat reintroduction. On the y‐axis, the patient's weight is shown, while the x‐axis represents the day of life (DOL). Key points are highlighted, including the initiation of feeding with skimmed milk, the start of fat reintroduction, the moment when a concentration of 1 g/dL is reached, and the point at which 2.8 g/dL is achieved.

The formula was applied to a premature infant with congenital chylothorax. The patient, delivered at 30 + 4 weeks, had bilateral pleural effusions and hydrops. Initial management included thoracocentesis and parenteral nutrition due to high pleural output (> 300 mL/day). On Day 17, defatted maternal milk (0.008 g/dL fat) was initiated with MCT, protein fortification, and fat‐soluble vitamins. Fat reintroduction with fresh maternal milk (fat content ranging 1.9–3 g/dL) began on Day 27, cautiously once chylothorax had resolved, using the formula, starting with 0.2 g/dL (< 10% of target fat). Fat content was increased in 0.2 g/dL every 2 days to reach 1 g/dL after 1 week and then to 2.5–3 g/dL, monitoring tolerance and confirming absence of chylothorax recurrence via serial thoracic ultrasounds. MCT supplementation was gradually withdrawn with normal growth (Figure 2). Breastfeeding was maintained for 12 months. No recurrence of chylothorax was observed.

## 4 DISCUSSION

Feeding defatted human milk is nutritionally beneficial for infants with chylothorax.^8^, ^9^, ^10^, ^11^, ^12^ It has been shown to be as safe as MCT‐based formula feeding in terms of chylothorax recurrence, with comparable growth outcomes when adequate nutritional supplementation is provided.^9^, ^10^, ^11^, ^12^ However, protocols for its use still leave unresolved questions, which we have attempted to address in this study.

Our study confirmed that standardized defatting methods^8^, ^16^ achieved near‐complete fat removal. This allows human milk to be safely administered during early stages of chylothorax, even when pleural output is still active but did not exceed 20 mL/kg/day.^12^ In addition, we have described several factors affecting residual fat, including centrifugation force,^8^, ^16^ baseline fat content and aliquot size, not previously described. Baseline fat content was higher in mothers of preterm infants or with prolonged lactation (> 6 months).

Those factors among others, like avoiding the use of hindmilk,^17^ should be considered when milk samples are selected for defatting to achieve near‐zero fat concentration. Another alternative to reach this is to perform successive centrifugations.

For nutritional analysis FTIR spectroscopy, widely used in the dairy industry, was employed. FTIR spectroscopy is recommended for accurate monitoring.^13^, ^14^ Moreover, FTIR allows for broader nutritional analysis beyond fat content, which enabled us to confirm that total protein content is not significantly altered by centrifugation, contrary to findings suggested by some authors.^18^ Some studies indicate that potential decreases in certain protein components may be more influenced by centrifugation force than duration.^19^ Nonetheless, to account for any possible protein loss adhering to the fat layer, human milk was appropriately fortified with protein particularly relevant in our case due to protein loss associated with chylothorax and prematurity.^20^ The approach proposed in this study represents one of the many applications enabled by FTIR technology in milk banks and neonatal units, facilitating a more personalized approach to neonatal nutrition.

The main contribution is a formula‐based method for fat reintroduction not previously described. The developed mathematical formula facilitates progressive and controlled increments, reducing the risk of chylothorax recurrence. Additionally, it facilitates individualization based on maternal (or donor, if applicable) milk composition, enhancing both safety and nutritional adequacy for the infant.

Clinical application showed no recurrence of chylothorax, successful, prolonged breastfeeding and adequate growth during and after the neonatal period especially after fat reintroduction. An initial conservative strategy reintroducing approximately 10% of the target fat intake, with subsequent gradual increases based on clinical tolerance and drainage response was established. MCT and protein supplementation during the fat‐free phase ensured acceptable growth during that period. Weight gain was also facilitated by the cessation of lymphatic loss and recovery from physiological preterm weight loss. We should consider the limitation that the validation of this approach is currently based on a single clinical application, and therefore its generalizability remains to be established in larger cohorts. Therefore, these findings should be extrapolated with caution when applied to other settings due the potential variability in maternal milk composition and possible differences in centrifugation protocols between centers.

## 5 CONCLUSION

The formula supports safe, individualized reintroduction of fat in infants with chylothorax, maintaining immunological and nutritional benefits of human milk. Further studies are needed, given the variability described in clinical practice,^12^, ^15^ to define optimal daily fat increments. Our formula provides a framework for future clinical research.

## CONFLICT OF INTEREST STATEMENT

The authors declare no conflicts of interest.

## References

[1] Attar MA , Donn SM . Congenital chylothorax. Semin Fetal Neonatal Med. 2017;22(4):234‐239. 10.1016/j.siny.2017.03.005 28351595

[2] Samanidis G , Kourelis G , Bounta S , et al. Postoperative chylothorax in neonates and infants after congenital heart disease surgery. Nutrients. 2022;14(9):1803. 10.3390/nu14091803 35565771 PMC9104302

[3] Resch B , Sever Yildiz G , Reiterer F . Congenital chylothorax of the newborn: a systematic analysis of published cases between 1990 and 2018. Respiration. 2022;101(1):84‐96. 10.1159/000518217 34515211 PMC8820433

[4] Santambrogio L . The lymphatic fluid. Int Rev Cell Mol Biol. 2018;337:111‐133. 10.1016/bs.ircmb.2017.12.002 29551158

[5] Bellini C , Cabano R , De Angelis LC , et al. Octreotide for congenital and acquired chylothorax in newborns: a systematic review. J Paediatr Child Health. 2018;54(8):840‐847. 10.1111/jpc.13889 29602276

[6] Biewer ES , Zürn C , Arnold R , et al. Chylothorax after surgery on congenital heart disease in newborns and infants: risk factors and efficacy of MCT‐diet. J Cardiothorac Surg. 2010;5:127. 10.1186/1749-8090-5-127 21144029 PMC3009966

[7] You YQ , Ling PR , Qu JZ , Bistrian BR . Effects of medium‐chain triglycerides, long‐chain triglycerides, or 2‐monododecanoin on fatty acid composition in the portal vein, intestinal lymph, and systemic circulation in rats. JPEN J Parenter Enteral Nutr. 2008;32(2):169‐175. 10.1177/0148607108314758 18407910 PMC3202979

[8] Concheiro‐Guisan A , Alonso‐Clemente S , Suarez‐Albo M , Duran‐Fernandez Feijoo C , Fiel‐Ozores A , Fernandez‐Lorenzo JR . The practicality of feeding defatted human milk in the treatment of congenital chylothorax. Breastfeed Med. 2019;14(9):648‐653. 10.1089/bfm.2019.0100 31403320

[9] Kocel SL , Russell J , O'Connor DL . Fat‐modified breast milk resolves chylous pleural effusion in infants with postsurgical chylothorax but is associated with slow growth. JPEN J Parenter Enteral Nutr. 2016;40(4):543‐551. 10.1177/0148607114566464 25560680

[10] Neumann L , Springer T , Nieschke K , Kostelka M , Dähnert I . ChyloBEST: chylothorax in infants and nutrition with low‐fat breast milk. Pediatr Cardiol. 2020;41(1):108‐113. 10.1007/s00246-019-02230-z 31729543

[11] DiLauro S , Russell J , McCrindle BW , Tomlinson C , Unger S , O'Connor DL . Growth of cardiac infants with post‐surgical chylothorax can be supported using modified fat breast milk with proactive nutrient‐enrichment and advancement feeding protocols; an open‐label trial. Clin Nutr ESPEN. 2020;38:19‐27. 10.1016/j.clnesp.2020.05.001 32690156

[12] Lion RP , Winder MM , Amirnovin R , et al. Development of consensus recommendations for the management of post‐operative chylothorax in paediatric CHD. Cardiol Young. 2022;32(8):1202‐1209. 10.1017/S1047951122001871 35792060

[13] Perrin MT , Festival J , Starks S , Mondeaux L , Brownell EA , Vickers A . Accuracy and reliability of infrared analyzers for measuring human milk macronutrients in a milk bank setting. Curr Dev Nutr. 2019;3(11):nzz116. 10.1093/cdn/nzz116 31723725 PMC6838652

[14] Andreassa NP , Suano‐Souza FI , Sarni ROS . Fat content and energy calculation in pasteurized human milk: comparison between infrared analysis and creamatocrit method. Breastfeed Med. 2024;19(11):863‐869. 10.1089/bfm.2024.0249 39263766

[15] Marino LV , Bell KL , Woodgate J , Doolan A . British Dietetic Association Paediatric Cardiology Interest Group. An international survey of the nutrition management of chylothorax: a time for change. Cardiol Young. 2019;29(9):1127‐1136. 10.1017/S1047951119001525 31414980

[16] Drewniak MA , Lyon AW , Fenton TR . Evaluation of fat separation and removal methods to prepare low‐fat breast milk for fat‐intolerant neonates with chylothorax. Nutr Clin Pract. 2013;28(5):599‐602. 10.1177/0884533613497763 23921298

[17] Pawelczyk M , Werner PL , Nielsen BW , Dąbrowski FA . Is increased fat content of hindmilk due to the size or the number of milk fat globules? Int Breastfeed J. 2009;4(7). 10.1186/1746-4358-4-7 PMC2717917 19607695

[18] Drewniak M , Waterhouse CCM , Lyon AW , Fenton TR . Immunoglobulin A and protein content of low‐fat human milk prepared for the treatment of chylothorax. Nutr Clin Pract. 2018;33(5):667‐670. 10.1177/0884533617722762 29730893

[19] Gharbi N , Marciniak A , Perreault V , Stone D , Fittipaldi N , et al. The effect of pasteurization treatment and skimming conditions on human milk proteins. LWT‐Food Sci Technol. 2022;172:114184. 10.1016/j.lwt.2022.114184

[20] Arslanoglu S , Moro GE , Ziegler EE . Fortification of human milk for preterm infants: update and recommendations. J Perinatol. 2019;39(1):41‐51. 10.1038/s41372-018-0266-4

